# graphite - a Bioconductor package to convert pathway topology to gene network

**DOI:** 10.1186/1471-2105-13-20

**Published:** 2012-01-31

**Authors:** Gabriele Sales, Enrica Calura, Duccio Cavalieri, Chiara Romualdi

**Affiliations:** 1 Department of Biology, University of Padova, via U. Bassi 58/B, Padova, Italy; 2 Department of Computational Biology, Istituto Agrario di San Michele all'Adige, Trento, Italy

## Abstract

**Background:**

Gene set analysis is moving towards considering pathway topology as a crucial feature. Pathway elements are complex entities such as protein complexes, gene family members and chemical compounds. The conversion of pathway topology to a gene/protein networks (where nodes are a simple element like a gene/protein) is a critical and challenging task that enables topology-based gene set analyses.

Unfortunately, currently available R/Bioconductor packages provide pathway networks only from single databases. They do not propagate signals through chemical compounds and do not differentiate between complexes and gene families.

**Results:**

Here we present graphite, a Bioconductor package addressing these issues. Pathway information from four different databases is interpreted following specific biologically-driven rules that allow the reconstruction of gene-gene networks taking into account protein complexes, gene families and sensibly removing chemical compounds from the final graphs. The resulting networks represent a uniform resource for pathway analyses. Indeed, graphite provides easy access to three recently proposed topological methods. The graphite package is available as part of the Bioconductor software suite.

**Conclusions:**

graphite is an innovative package able to gather and make easily available the contents of the four major pathway databases. In the field of topological analysis graphite acts as a provider of biological information by reducing the pathway complexity considering the biological meaning of the pathway elements.

## 1 Background

A great deal of effort has been recently directed towards the study of gene sets (hereafter GSA) in the context of microarray data analysis. The aim is to identify groups of functionally related genes with possibly moderate, but coordinated, expression changes. Several GSA tests, both univariate and multivariate, have been recently developed. See [1] for a comprehensive review, and [2-4] for a detailed description and a critical investigation of the tested hypotheses.

These approaches, although effective, miss the information of the topological properties of the pathways. To this end, the seminal paper by Draghici et al. [5] proposed a radically different approach (called impact analysis, SPIA) attempting to capture several aspects of the data: the fold change of differentially expressed genes (DEGs), the pathway enrichment and the topology of signaling pathways. In particular, SPIA enhances the impact of a pathway if the DEGs tend to lie near its entry points. Massa et al. [6] introduced an alternative approach that is based on a correlation structure test. Specifically, the graphical model theory is used to decompose the overall pathway into smaller cliques, with the aim of exploring in detail small portions of the entire model. Recently, Isci et al. [7] proposed a Bayesian Pathway Analysis that models each biological pathway as a Bayesian network (BN) and considers the degree to which observed experimental data fits the model. Finally, Laurent et al. [8] developed a graph-structured two-sample test of means for problems in which the distribution shift is assumed to be smooth on a given graph.

In this perspective the retrieval of pathway information and the subsequent conversion into a gene/protein network is crucial. However, pathway annotations comprise a myriad of interactions, reactions, and regulations which is often too rich for the conversion to a network. In particular, challenges are posed by the presence of chemical compounds mediating interactions and by different types of gene groups (e.g. protein complexes or gene families) that are usually represented as single nodes. Available R packages (KEGGgraph, [9] and NCIpath) share some drawbacks: i) they are focused on a single pathway database each; ii) they do not consider gene connections through chemical compounds; iii) they do not handle differently the various kinds of biological gene groups.

Here we present graphite (GRAPH Interaction from pathway Topological Environment) a Bioconductor package that provides networks from the pathways of four databases (Biocarta; KEGG, [10]; NCI/Nature Pathway Interaction Database, [11]; Reactome, [12]). It discriminates between different types of biological gene groups; propagates gene connections through chemical compounds; allows the selection of edges by type of interaction; uniformly converts heterogeneous node IDs to EntrezGene IDs and HUGO symbols; and finally allows the user to directly run SPIA, DEGraph, and topologyGSA analyses over the provided networks.

## 2 Implementation

graphite was implemented using the statistical programming language R and the package is included in the open-source Bioconductor project [13]. In section 2.1 we report a brief state of the art of pathway formats, databases and tools, while in section 2.2 we report the rules that graphite uses to convert pathway topology to gene networks.

### 2.1 Pathways Background

A variety of databases containing information on cell signaling pathways have been developed in conjunction with methodologies to access and analyse the data [14]. Pathway databases serve as repositories of current knowledge on cell signaling. They present pathways in a graphical format comparable to the representation present in text books, as well as in standard formats allowing the exchange between different software platforms and further processing by network analysis, visualization and modeling tools. At the present day, there exist a vast variety of databases containing biochemical reactions, such as signaling pathways or protein-protein interactions. The Pathguide resource serves as a good overview of current pathway databases [15]. It lists more than 200 pathway repositories; over 60 of those are specialized on reactions of the human species. However, only half of them provide pathways and reactions in computer-readable formats needed for automatic retrieval and processing, such as Biological Pathway Exchange (BioPAX, [16]), Systems Biology Markup Language (SBML, [17]) and Biological Connection Markup Language (BCML, [18]). Thus, different databases are characterised by different annotations and only a part of the whole set of reactions are confirmed by all the repositories. On the other hand, Cerami et al. [19] have recently developed a web repository aiming at collecting and integrating all public pathway data available in standard formats. It currently contains data from nine databases with over 1400 pathways and 687,000 interactions.

From the graphical point of view, a number of software tools [20-28] have been developed to visually build computable models of pathways. For additional details see the web page http://www.sbgn.org/. These tools are usually based on graphical models in which nodes represent genes, proteins or chemical compounds, and edges represent various types of interactions or associations.

In order to gather curated pathway data we collect pathway information from the three public databases that have emerged as reference points for the system biology community. Reactome [12] (data was retrieved in the BioPax format from the Reactome web site), backed by the EBI, is one of the most complete repositories; it is frequently updated and provides a semantically rich description of each pathway. KEGG Pathways [10] (retrieved in KGML format) provides maps for both signalling and metabolic pathways, supplemented by 19 highly interconnected databases with genomic, chemical and phenotypic information. BioCarta (http://www.biocarta.com) and NCI (NCI/Nature Pathway Interaction Database) [11] whose data were retrieved in BioPax format from the PDI database web page.

### 2.2 Pathway topology conversion to gene network

Pathway topologies converted to gene-gene networks (simple interaction format, hereafter SIF) is available either on the Reactome [12] or on Pathway Commons [19] web sites. However, they provide a single huge file of protein-protein reaction for each database.

**Figure 1:**
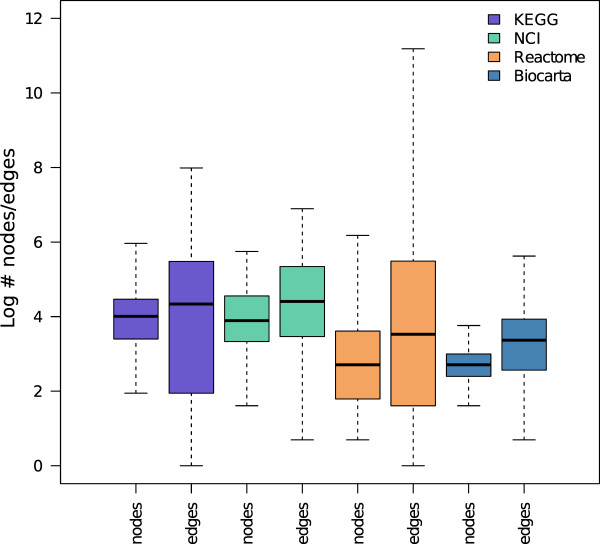
**Edges and nodes distribution of networks after pathway conversion according to the selected database**.

Unfortunately topological pathway analysis (TPA) methods are not based on the analysis of the interaction network as a whole, but needs a separate graph for each pathway, in order to identify those that are significantly involved in the biological problem under investigation. Moreover, TPA benefits of long paths in which the gene signal can spread across compounds and cell compartments. Paxtools, a Java library for working with BioPAX (http://www.biopax.org/paxtools.php), defines some rules to convert a BioPax file to a SIF. However, it does not take into account signal compound propagation and gene group expansion. We start from Paxtools rules and we extend them in order to reduce both BioPAX and KGML interactions to pairwise relationships with compound mediated signal propagation. Table 1 and Figure 1 report respectively pathway summary statistics and nodes/edges distributions for the four databases obtained after the conversion. In the following we describe in detail some of our rules.

**Table 1:** Number of pathways converted to networks with average number of edges and nodes according to the selected database.

Database	N. of pathways	Mean (Median) nodes	Mean (Median) edges
KEGG	232	71.86 (54)	211.12 (75.5)
Reactome	1070	33.22 (14)	780.64 (33)
BioCarta	254	15.18 (14)	36.88 (28)
NCI	177	76.79 (48)	165.18 (81)

#### 2.2.1 Pathway definition

The KEGG database provides separate xml files, one for each pathway. In the the other databases, we consider as a pathway every instance of the BioPax class pathway.

#### 2.2.2 Nodes with multiple elements

**Figure 2:**
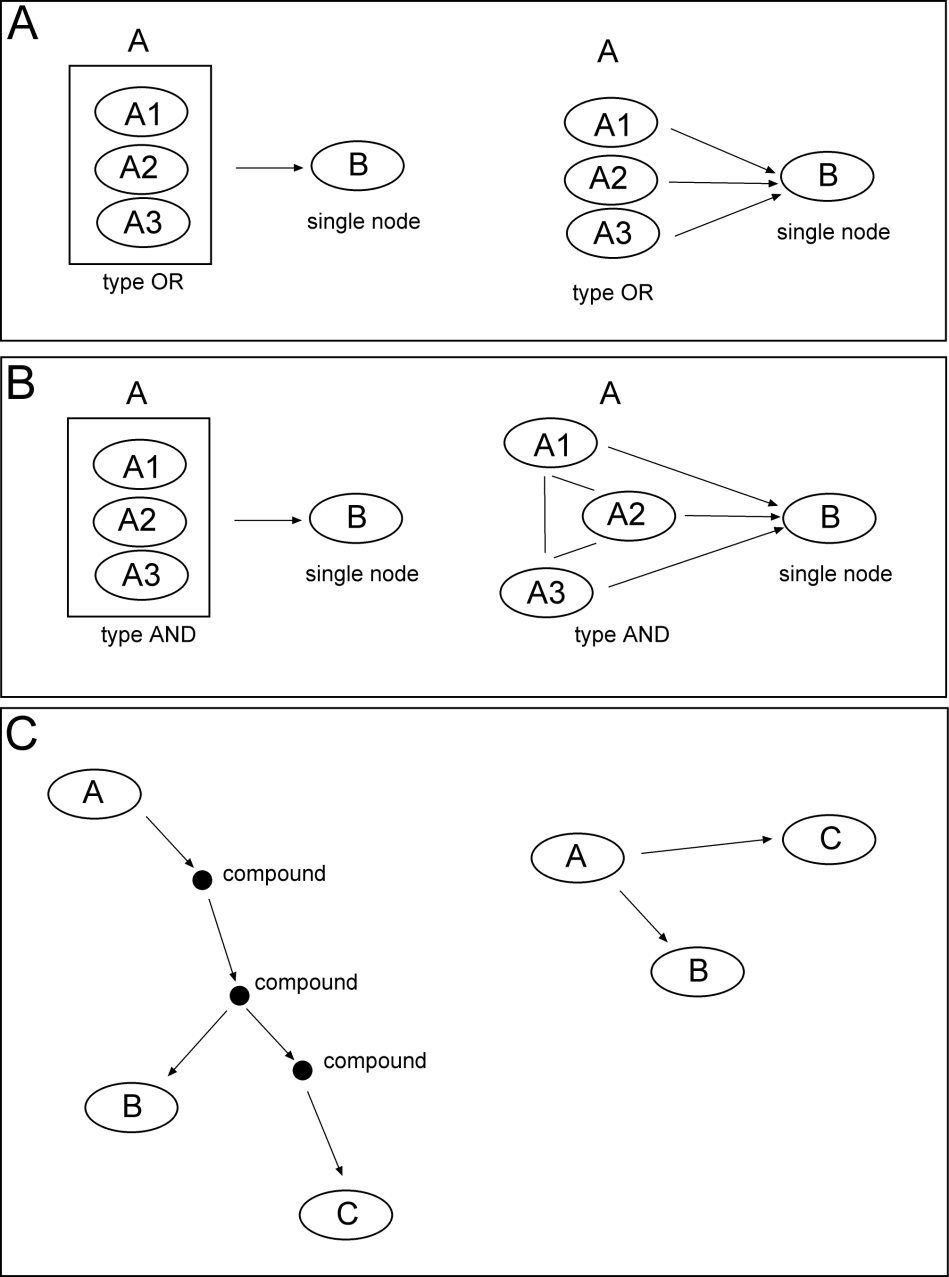
**Toy examples of nodes with multiple elements converted to gene-network**. Group AND (protein complexes, Panel A), group OR (member of gene family, Panel B) and compound mediated signal (panel C).

Within a pathway, nodes often correspond to multiple gene products. These can be divided into protein complexes (proteins linked by protein-protein interactions) and groups containing alternative members (like gene families, genes with similar biochemical functions). These groups should be considered differently: the first kind (hereafter group AND) should be expanded into a clique (all proteins connected to the others), while the second (hereafter group OR) should be expanded without connections among them see Figure 2 (panel A and B).

In the KGML format there are two ways of defining nodes with multiple elements: protein complexes (group AND defined by entry type = "group", see Figure 2A) and groups with alternative members (group OR defined by entry type = "gene", see Figure 2B). An example of the KGML for AND and OR groups can be seen in Additional file 1.

BioPax allows only one type of group: protein complexes (group AND) corresponding to the complex class. However, it often happens that a protein instance contains multiple xrefs pointing to alternative elements of the process (group OR). An example of BioPax definitions for both the AND and the OR groups can be seen in Additional file 1.

#### 2.2.3 Compound mediated interactions

Compound-mediated interactions are interactions for which a compound acts as a bridge between two elements (see Figure 2C). As chemical compounds are not usually measured with high-throughput technologies, they should be removed from the network. However, the trivial elimination of the compounds will strongly bias the topology interrupting the signals passing through them. If element *A *is linked to compound *c *and compound *c *is linked to element *B*, then *A *should be linked to *B*. Moreover, to best fit the biological model we take into account cell compartment membership: the connection among genes *A *and *B *is established only if the shared compound *c *has the same localization in both the reactions. However, there are some chemical compounds that are highly frequent in map description (such as hydrogen, *H*_2_*O*,...). Signal propagation through them would lead to degenerate and long chains of compounds. To remove this artifact, we decided to ignore these compounds during the signal propagation. After parsing all the BioPax and KGML data we obtain compound chains whose distribution are reported in Table 2.

**Table 2:** Frequency of compound chains that we propagate according to different databases.

Chain length	KEGG	Reactome	Biocarta	NCI
2	19790	55155	502	2790
3	0	874	9	134
4	0	736	8	11
5	0	140	0	0
6	0	39	0	0
7	0	6	0	0
8	0	17	0	0
9	0	1	0	0

Within the KGML format there are two different ways of describing a compound mediated interaction: i) direct interaction type = "PPrel" (*A *interacts to *B *through compound *c) *and ii) indirect one type = "PCrel" (*A *interacts to compound *c *and *c *interacts to *B*).

The BioPax format, on the other hand, provides only an indirect way of defining compound mediated signals (see Additional file 1).

#### 2.2.4 Relation attributes

graphite allows the user to see the single/multiple relation types that characterize an edge. The edge types have been kept as close as possible to those annotated in the original formats. Some new types have been introduced due to the needs of the topological conversion.

## 3 Results and discussion

In sections 3.1 and 3.2 we provide two examples of pathway topologies converted to gene networks by graphite, while in section 3.3 we show the core functions to retrieve, convert and display graphite networks. In section 3.4 we perform a simulation study to verify the efficacy of our signal propagation strategy in terms of topological analyses. In section 3.5 we run an example of topological gene set analysis using graphite networks and in section 3.6 we critically compare graphite with other available R/Bioconductor packages providing pathway topologies.

### 3.1 Pathway conversion example 1: Insulin signaling pathway

**Figure 3:**
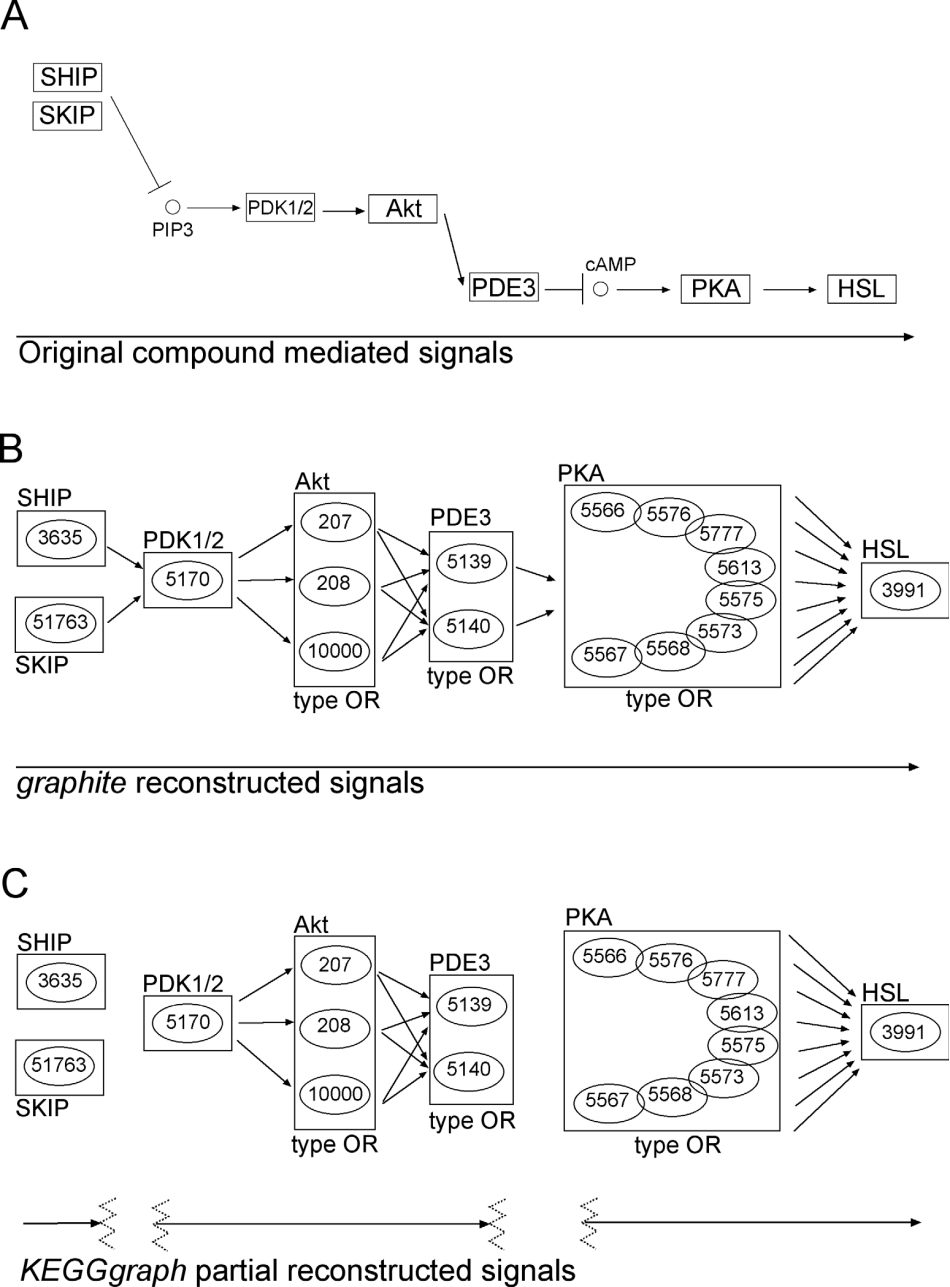
**Differences in signal reconstruction of a selected portion of the insulin signaling pathway of KEGG (hsa04910)**. Panel A. The original signal cascade. Panel B. graphite signal reconstruction through chemical compound propagation. Numbers represent EntrezGene IDs. Panel C. KEGGgraph signal reconstruction.

Figure 3 represent an example in which the simple elimination of compounds leads to an incorrect network topology.

Insulin is an hormone controlling the balance between mobilization and storage of energy molecules. Insulin binds the Insulin Receptor (IR) and through phosphorilation of the IRS adaptors is able to recruit and activate PI3K. PI3K is a kinase that converts PIP2 in PIP3 which is a secondary messenger involved in the regulation of various processes. The conversion between PIP3 into PI(3,4)P2 or PI(4,5)P2 operated by phosphatases like SHIP1/2 or PTEN induce a depletion of PIP3 levels and of consequence a reduced activity on its downstream targets [29].

PIP3 associates with the inner lipid bilayer of the plasma membrane to promote the recruitment of proteins with pleckstrin homology (PH) domains, like PDPK and AKT, which is a crucial mediator of various cell process, such as apoptosis, cell cycle, protein synthesis, regulation of metabolism [30].

Among other functions, AKT activates also the cyclic nucleotide phosphodiesterases (PDEs), that is a group of enzymes able to regulate the localization, duration, and amplitude of the cyclic nucleotides. Signaling PDEs are therefore important regulators of signal transduction mediated by these second messenger molecules [31]. In this pathway, PDE, depleting cAMP, indirectly inhibits the PKC mediated phosphorilation, and the activation of LIPE that is a lipase able to mobilize lipid energy stores. PDE acts, in this way, as a anti-lipolytic agents [32].

This hormonal mediated signaling cascade, from the insulin receptor to the inhibition of HSL, involves two "second messenger" compounds (PIP3 and cAMP) crucial for the transduction of the signal.

In panel A of Figure 3 we report a part of the insulin signaling pathway of KEGG (hsa4910) that contains three groups OR (PDE3, AKT and PKA), and two compound mediated interactions (through PIP3 and cAMP). This is a clear examples of a signal cascade in which the propagation of the signal through compounds is crucial to keep the whole signaling path. In panel B we report graphite reconstructed signal cascade while in panel C the KEGGgraph partially reconstructed signal.

An extract of the xml file corresponding to the signal reported in Figure 3 of the main text is present in Additional file 1. From the xml definition it is evident how entry 2 (SKIP) and entry 3 (SHIP) are linked to compound 15 (PIP3) while there is no direct interaction between compound 15 (PIP3) and entry 62 (PDK1/2). This is why KEGGgraph misses the signal, while graphite captures it by splitting the relation between entry 52 (protein complex P13K) and 62 (PDK1/2) through compound 15 (PIP3) into both 52 to 15 and 15 to 62. This dissection allows the reconstruction of the signal, otherwise impossible.

### 3.2 Pathway conversion example 2: catalysis and cleavage of Notch 1 by Gamma Secretase Complex

We selected the reaction 1784.3 from the Reactome pathway called "A third proteolytic cleavage releases NICD". Gamma secretase is a multi-subunit protease complex, itself an integral membrane protein, that cleaves single-pass transmembrane proteins at residues within the transmembrane domain. Here represented the processing of the Notch 1 protein. The gamma-secretase complex is composed of Presenilin homodimer (PSEN1 variant 1 or 2 or 3 or 4 or 5 and PSEN2 variant 1 or 2), Nicastrin (NCSTN variant 1 or variant 2), APH1 (APH1A or APH1B) and PEN2. Maturation of the Notch receptor involves a cleavage of the protein, the intracellular domain is liberated from the plasma membrane that can enter into the nucleus to engage other DNA-binding proteins regulating gene expression. The cleavage is catalyzed and performed by Gamma Secretase Complex.

**Figure 4:**
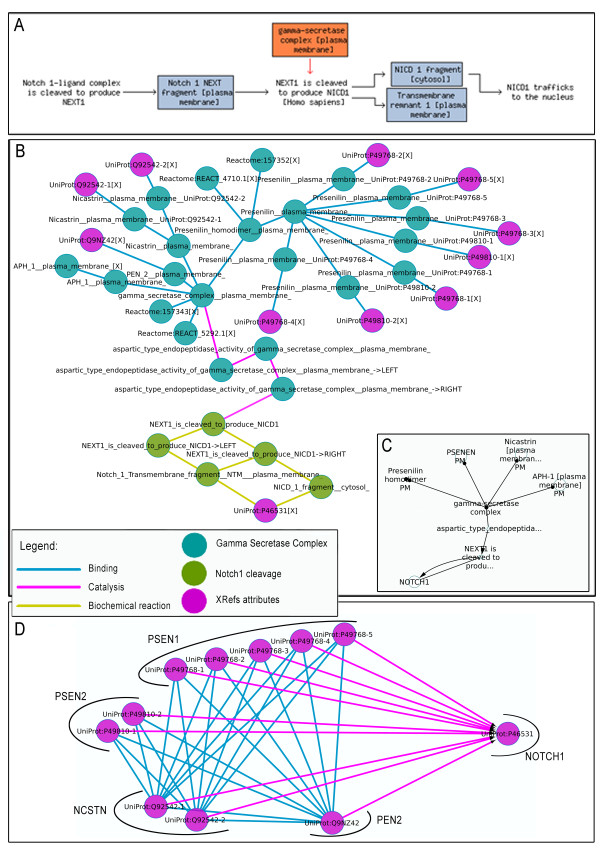
**Catalysis and cleavage of Notch 1 by Gamma Secretase Complex**. Reactome representation of the reactions (Panel A), BioPax information as it is stored in owl model and in Cytoscape plug-in BioPax dedicated (respectively panel B and C) and the graphite final network (panel D).

Figure 4 shows Reactome representation of the reactions (Panel A), the BioPax information as it is stored in owl model and in Cytoscape plug-in for BioPax (respectively panel B and C) and the graphite final network (panel D). In the graphite network the nodes are annotated using the XRefs informations while edges preserve the type of the reaction annotated the OWL model. Distinction between OR complexes (formed by all the possibile variants of each protein) nested inside the AND complex of the Gamma secretase are topologically preserved in the resulting graph.

### 3.3 graphite functions

To access the Reactome, KEGG, Biocarta and NCI databases graphite uses respectively the lists reactome, kegg, biocarta and nci. A pathway network can be retrieved from one of the lists using the name of the pathway,

> names(biocarta)[1:3]

[1] "acetylation and deacetylation of rela in nucleus"

[2] "actions of nitric oxide in the heart"

[3] "activation of camp-dependent protein kinase pka"

> biocarta[["ras signaling pathway"]]

"ras signaling pathway" pathway from BioCarta

Number of nodes = 18

Number of edges = 22

Type of identifiers = native

Retrieved on = 2011-05-12

or its position in the list of pathways:

> p <- biocarta [[175]]

> p@title

[1] "ras signaling pathway"

In the network, nodes represent genes and edges functional relationships among them. Nodes can have heterogeneous IDs (according to the pathway original annotation) and edges can be characterized by multiple functional relationships.

The function pathwayGraph builds a graphNEL object from a pathway p:

> g <- pathwayGraph(p)

> g

A graphNEL graph with directed edges

Number of Nodes = 18

Number of Edges = 23

> edgeData(g) [1]

$ 'EntrezGene:10928 | EntrezGene:5879'

$ 'EntrezGene: 10928 | EntrezGene:5879 '$weight

[1] 1

$ 'EntrezGene:10928 | EntrezGene:5879 '$edgeType

[1] "catalysisOut (ACTIVATION)"

The function converterIdentifiers allows the user to map such variety of IDs to a single type (Entrez-Gene or Gene Symbol). For the ID conversion graphite uses the data provided by the Bioconductor package org.Hs.eg.db. This mapping process, however, may lead to the loss of some nodes (not all identifiers may be recognized) and has an impact on the topology of the network (one ID may correspond to multiple IDs in another annotation or vice versa).

> pEntrez <- convertIdentifiers (p, "entrez")

> pEntrez

"ras signaling pathway" pathway from BioCarta

Number of nodes = 20

Number of edges = 20

Type of identifiers = Entrez Gene

Retrieved on = 2011-05-12

> nodes(pEntrez)[1:10]

[1] "10928" "1147" "3265" "387" "4303" "5295" "572" "5879" "5894" "5898"

Several pathways have a huge number of nodes and edges, thus there is the need of an efficient system of visualization. To this end graphite uses the Rcytoscape package to export the network to Cytoscape [27]. Cytoscape is a Java based software specifically built to manage biological network complexity and for this reason it is widely used by the biological community. Run Cytoscape with RPC plugin enabled and type at the R command prompt:

> cytoscapePlot(convertIdentifiers(a$'toll-like receptor pathway', "symbol"))

**Figure 5:**
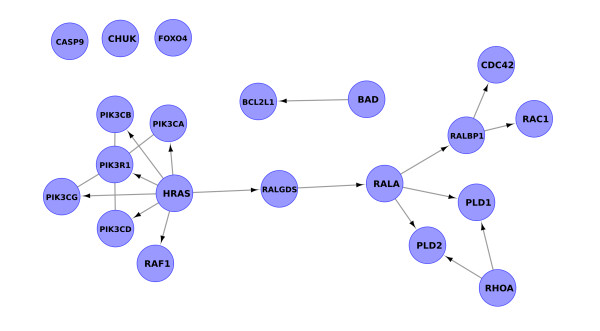
**Visualization of **graphite **network using RCytoscape package**.

The network will be automatically loaded into Cytoscape. See Figure 5 for the result of this operation.

### 3.4 Simulation study: compound propagated signal improves topological analysis

**Figure 6:**
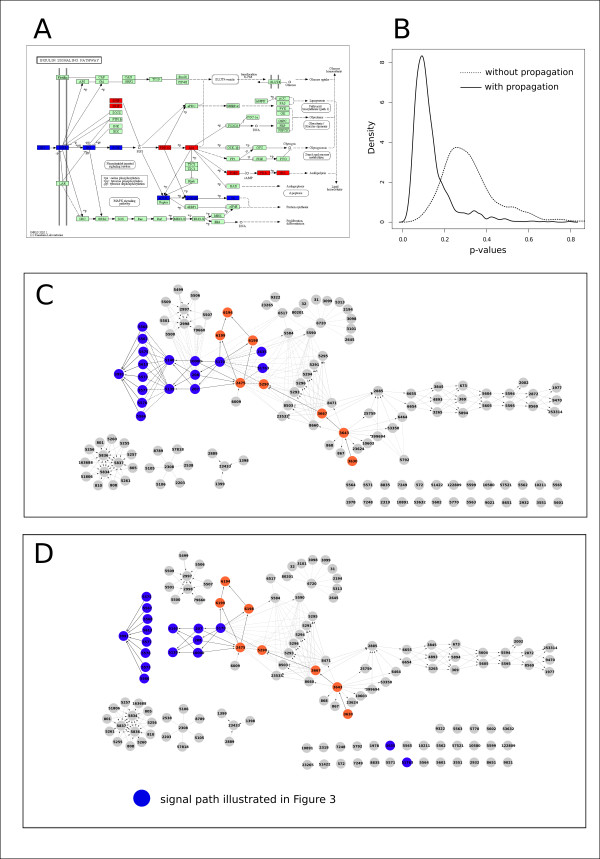
**Results of the simulation study on the Insulin signaling pathway compound mediated signal propagation**. Panel A. Signal paths selected to be differentially expressed. Panel B. p-value distribution of the topological analysis SPIA (pPERT) with and without propagation. Panel C. graphite network obtained from insulin pathway with propagation. Panel D. network obtained from insulin pathway without propagation.

In order to verify our signal propagation strategy we perform a simulation study. Using the insulin signaling pathway of the KEGG database we select as differentially expressed 22 genes lying on the signal paths highlighted in Figure 6A. These genes are connected if propagation is employed, otherwise they are disconnected (see Figure 6C and 6D for propagation and non-propagation respectively). We expect that propagation will lead to better results in terms of topological analyses.

Our simulation is based on the following steps: 1) we randomly generate *μ_FC _*~ *U *(2, 10); 2) we randomly generate log fold change values (*δ_i _*for *i *= 1,..., 22) of the differentially expressed genes as *δ_i _*~ *N *(*μ_FC_*, 2) (interactions of the signal paths selected are characterized all by activation, thus, fold changes have the same sign); 3) we run the SPIA[5] algorithm on the Insulin signaling pathway with and without signal propagations and we take the p-value of the topological analysis (pPERT); 4) we repeat from step 1 10,000 times.

As shown in Figure 6B the distribution of the topological significance p-values in case of signal propagation is shifted towards lower values with respect to the case of non-propagation. Propagation p-value distribution is not only centered on 0.1 (while the one with non-propagation is centered on 0.3) but is also less variable. As expected the same results are obtained simulating negative fold changes (data not shown). This finding demonstrate that compound mediating signal propagation improves topological analyses giving more reliable results.

### 3.5 Example of topological analysis: B-lineage Adult Acute Lymphocytic Leukemia

#### 3.5.1 Data

The dataset, recently published by [33], characterizes gene expression signatures in acute lymphocytic leukemia (ALL) cells associated with known genotypic abnormalities in adult patients. Several distinct genetic mechanisms lead to acute lymphocytic leukemia (ALL) malignant transformations deriving from distinct lymphoid precursor cells that have been committed to either T-lineage or B-lineage differentiation. Chromosome translocations and molecular rearrangements are common events in B-lineage ALL and reflect distinct mechanisms of transformation. The relative frequencies of specific molecular rearrangements differ in children and adults with B-lineage ALL. The BCR breakpoint cluster region and the c-abl oncogene 1 (BCR/ABL) gene rearrangement occurs in about 25% of cases in adult ALL, and much less frequently in pediatric ALL.

Data is available at the Bioconductor site (http://www.bioconductor.org/help/publications/2003/Chiaretti/chiaretti2/) Expression values, appropriately normalized according to rma and quantile normalization, derived from Affymetrix single channel technology, consist of 37 observations from one experimental condition (*n*_1 _= 37, BCR; presence of BCR/ABL gene rearrangement) and 41 observations from another experimental condition (*n*_2 _= 41, NEG; absence of rearrangement). Probes platform have been annotate using EntrezGene custom CDF version 14 [34]. Given the involvement of BCR and ABL genes in the chimera rearrangement, we expect these genes playing a central role in the gene set analysis; thus, most of the pathways containing BCR and/or ABL genes should be found as significant.

#### 3.5.2 Results

We report the results obtained by SPIA[5] and topologyGSA[6] on the graphite networks. These statistical tests are based on completely different null hypotheses; while SPIA needs the list of differentially expressed genes, topologyGSA performs two statistical tests (to compare the mean and the variance of the pathway between two groups) on the entire list of genes belonging to a pathway. Here, differentially expressed genes required for SPIA package have been identified using RankProd test [35] (*F D R <*0.01), while the test on the mean has been chosen for topologyGSA package.

Tables 3 and 4 reports the list of significant pathways identified by the above approaches; pathways marked with *✓ *are those containing BCR and/or ABL genes. It is interesting to observe that several pathways containing either BCR and ABL genes were identified as deregulated especially with topologyGSA. Then, as expected, several additional pathways associated to cancer progression, apoptosis, cell cycle, cell proliferation and inflammation have been selected as significant.

**Table 3:** Pathway analysis performed using SPIA statistical test on graphite networks.

	Name	FDR	Signal	Database	BCR	ABL
1	Leishmaniasis	0.03	Activated	KEGG		
2	Phase 1 - Functionalization of compounds	0.02	Activated	Reactome		
3	Syndecan-4-mediated signaling events	0.00	Activated	NCI		
4	Regulation of RAC1 activity	0.00	Activated	NCI		
5	RAC1 signaling pathway	0.00	Activated	NCI		
6	RhoA signaling pathway	0.00	Activated	NCI		
7	Regulation of RhoA activity	0.00	Activated	NCI		
8	Noncanonical Wnt signaling pathway	0.00	Activated	NCI		
9	Wnt signaling network	0.00	Activated	NCI		
10	BCR signaling pathway	0.00	Inhibited	NCI		
11	IL6-mediated signaling events	0.00	Inhibited	NCI		
12	Hypoxic and oxygen homeostasis regulation of HIF-1-alpha	0.00	Inhibited	NCI		
13	Stabilization and expansion of the E-cadherin adherens junction	0.00	Activated	NCI		
14	E-cadherin signaling in the nascent adherens junction	0.00	Activated	NCI		
15	E-cadherin signaling events	0.00	Activated	NCI		
16	HIF-1-alpha transcription factor network	0.00	Inhibited	NCI		
17	ALK1 signaling events	0.01	Activated	NCI		
18	Canonical Wnt signaling pathway	0.02	Activated	NCI		
19	ALK1 pathway	0.02	Activated	NCI		
20	S1P2 pathway	0.02	Inhibited	NCI		
21	Regulation of nuclear SMAD2/3 signaling	0.02	Activated	NCI		
22	Regulation of cytoplasmic and nuclear SMAD2/3 signaling	0.02	Activated	NCI		
23	TGF-beta receptor signaling	0.02	Activated	NCI		
24	C-MYB transcription factor network	0.02	Activated	NCI		
25	Osteopontin-mediated events	0.02	Inhibited	NCI		
26	Direct p53 effectors	0.02	Inhibited	NCI		
27	Validated transcriptional targets of AP1 family members Fra1 and Fra2	0.03	Activated	NCI		
28	Regulation of nuclear beta catenin signaling and target gene transcription	0.03	Activated	NCI		
29	S1P4 pathway	0.03	Inhibited	NCI		
30	amb2 Integrin signaling	0.03	Activated	NCI		
31	p38 MAPK signaling pathway	0.04	Activated	NCI		
32	Posttranslational regulation of adherens junction stability and dissassembly	0.04	Activated	NCI		
33	N-cadherin signaling events	0.04	Activated	NCI		
34	Lissencephaly gene (LIS1) in neuronal migration and development	0.05	Activated	NCI		*✓*
35	C-MYC pathway	0.06	Inhibited	NCI		
36	p53 pathway	0.06	Activated	NCI		

**Table 4:** Pathway analysis performed using topologyGSA statistical test on graphite networks.

	Name	FDR	Database	BCR	ABL
1	CDO in myogenesis	0.00	Reactome		*✓*
2	Regulation of cytoskeletal remodeling and cell spreading by IPP complex components	0.00	Reactome		
3	Role of Abl in Robo-Slit signaling	0.00	Reactome		*✓*
4	NF-kB activation through FADD/RIP-1 pathway mediated by caspase-8 and -10	0.01	Reactome		
5	TNF signaling	0.01	Reactome		
6	G1 Phase	0.02	Reactome		
7	mTOR signalling	0.02	Reactome		
8	PI3K Cascade	0.02	Reactome		
9	Cyclin D associated events in G1	0.02	Reactome		
10	PI-3K cascade	0.03	Reactome		
11	E2F mediated regulation of DNA replication	0.04	Reactome		
12	Cyclin A/B1 associated events during G2/M transition	0.04	Reactome		
13	Intrinsic Pathway for Apoptosis	0.04	Reactome		
14	Extrinsic Pathway for Apoptosis	0.05	Reactome		
15	Lissencephaly gene (LIS1) in neuronal migration and development	0.00	NCI		*✓*
16	ErbB4 signaling events	0.01	NCI		
17	Regulation of retinoblastoma protein	0.00	NCI		*✓*
18	Canonical NF-kappaB pathway	0.01	NCI		
19	p73 transcription factor network	0.01	NCI		*✓*
20	Atypical NF-kappaB pathway	0.02	NCI		
21	Neurotrophic factor-mediated Trk receptor signaling	0.00	NCI		*✓*
22	Pathogenic Escherichia coli infection	0.00	KEGG		*✓*
23	Chronic myeloid leukeamia	0.00	KEGG	*✓*	*✓*
24	Cell cycle	0.0	KEGG		*✓*
25	Axon guidance	0.00	KEGG		*✓*
26	Neurotrophin signaling pathway	0.00	KEGG		*✓*
27	mtor signaling pathway	0.01	Biocarta		
28	nf-kb signaling pathway	0.01	Biocarta		
29	tnf/stress related signaling	0.02	Biocarta		
30	p53 signaling pathway	0.03	Biocarta		
31	tnfr1 signaling pathway	0.02	Biocarta		
32	integrin signaling pathway	0.02	Biocarta		
33	erk and pi-3 kinase are necessary for collagen binding in corneal epithelia	0.02	Biocarta		
34	rb tumor suppressor/checkpoint signaling in response to dna damage	0.03	Biocarta		
35	egf signaling pathway	0.04	Biocarta		
36	tgf beta signaling pathway	0.04	Biocarta		
37	role of mitochondria in apoptotic signaling	0.04	Biocarta		
38	inhibition of cellular proliferation by gleevec	0.04	Biocarta		
39	atm signaling pathway	0.05	Biocarta		*✓*
40	influence of ras and rho proteins on g1 to s transition	0.05	Biocarta		

Leaving the comparison between topological analyses aside (because it is out of the scope of the present work), the results testify the feasibility of performing analyses using graphite and the ability to obtain reliable results independently of the chosen analysis method. In addition, for the first time, thanks to graphite all the topological methods gain the access to pathway repositories previously not considered.

Our results highlight that the hierarchical pathway structure and the reduced dimension of the pathways characterizing respectively the Reactome and Biocarta databases jointly with the specialized cancer pathways of the NCI databases allow the user to have deeper insight into the data.

To highlight the usefulness of topological analysis in the context of transcriptomic data interpretation, we report two graphite networks identified as significantly altered in the previous analysis.

**Figure 7:**
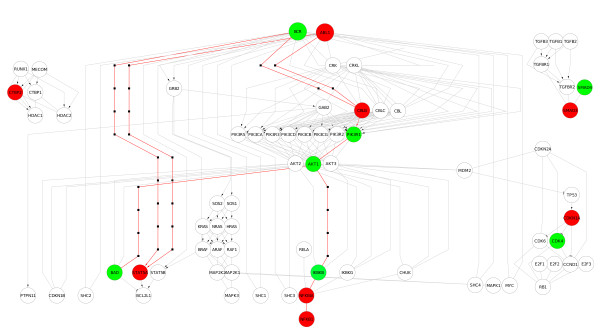
**Visualization of the chronic myeloid leukemia network of **graphite, **that contain BCR and ABL1 genes**. Colors represent up or down regulated genes between positive and negative BCR/ABL rearrangement.

Chronic myeloid leukemia pathway includes both genes, BCR and ABL1, and was identified as differentially expressed between BCR/ABL positive and negative patients by topologyGSA. Figure 7 shows the chronic myeloid leukemia graphite network from KEGG database with differentially expressed genes mapped with different colors according to fold change sign. It is interesting to note the presence of several OR groups (e.g. PI3K, AKT, IKK, CBL gene families), single members of which resulted to be differentially expressed. Two clear deregulated paths starting from BCR and ABL1 genes towards apoptosis and NFKB pathways highlight the power of topological analysis to deeper investigate signal cascades within large pathways.

### 3.6 R/Bioconductor packages

Currently there are two Bioconductor packages that try to convert pathway to the SIF model. KEGGgraph[9] that parses KGML format but i) considers all group of genes as groups OR and ii) completely removes compounds without propagation and NCIgraph that imports BioPax models from Cytoscape [27] without taking into account groups and compound propagation (compounds become nodes of the network) and uses internal IDs as node labels. The package allows to retain only nodes with EntrezGene IDs loosing all the other nodes. Thus, both of them are not suitable for topological pathway analyses.

## 4 Conclusions

It is evident that gene set analysis is moving towards considering pathway topology as a crucial feature. A correct conversion of the pathway topology to a gene network becomes therefore important. Available packages are not able to correctly reconstruct the signal transduction in most cases. graphite, on the other hand, is an innovative package able to gather and make easily available the contents of the four major pathway databases. In the field of topological analysis graphite acts as a provider of biological information by reducing the pathway complexity considering the biological meaning of the pathway elements.

## 5 Availability and requirements

• Project name: graphite

• Project home page: http://www.bioconductor.org/packages/devel/bioc/html/graphite.html • Operating system(s): Platform independent

• Programming language: R

• Other requirements: Bioconductor

• License: GNU AGPL

• Any restrictions to use by non-academics: none

## Authors' contributions

GS, EC and CR jointly define the concept proposed. GS, EC developed the methods proposed and performed the analysis on gene expression data. CR supervised the work and wrote the paper. DC Participated in the critical definition of the concept proposed and participated in drafting and commenting critically the manuscript. All Authors read and approved the final manuscript.

## Supplementary Material

Additional file 1 **Example of KGML and owl**. Additional file 1: bmc-supp.pdf, 116 K. http://www.biomedcentral.com/imedia/1501537976613594/supp1.pdf Click here for file
