# Supplementary material for: graphite - a Bioconductor package to convert pathway topology to gene network
Source: BMC Bioinformatics. 2012 Jan 31;13:20. doi: 10.1186/1471-2105-13-20 (PMC3296647; doi:10.1186/1471-2105-13-20)
Supplement: Additional file 1 — Example of KGML and owl. Additional file 1: bmc-supp.pdf, 116 K. http://www.biomedcentral.com/imedia/1501537976613594/supp1.pdf [file 1471-2105-13-20-S1.PDF]

graphite - a Bioconductor package to convert pathway topology to gene network

Gabriele Sales[1&], Enrica Calura[1&], Duccio Cavalieri[2] and Chiara Romualdi[*1]

[1]Department of Biology, University of Padova, via U. Bassi 58/B, Padova, Italy
[2]Department of Computational Biology, Istituto Agrario di San Michele all'Adige, Trento, Italy
[&] equally contributed

Email: Gabriele Sales - gabriele.sales@unipd.it; Enrica Calura - enrica.calura@unipd.it; Duccio Cavalieri - duccio.cavalieri@iasma.it; Chiara Romualdi - chiara.romualdi@unipd.it;

[*]Corresponding author

1 Supplementary material: groups AND - group OR

KGML for group AND and group OR:

```
Group AND:


     <entry id="1" name="hsa:7248" type="gene"
        link="http://www.kegg.jp/dbget-bin/www_bget?hsa:7248">
        <graphics name="TSC1, KIAA0243, LAM, MGC86987, TSC" ...
             type="rectangle" x="616" y="492" width="46" height="17"/>
    </entry>
     <entry id="40" name="hsa:7249" type="gene"
        link="http://www.kegg.jp/dbget-bin/www_bget?hsa:7249">
        <graphics name="TSC2, FLJ43106, LAM, TSC4" ...
             type="rectangle" x="616" y="475" width="46" height="17"/>
    </entry>
    <entry id="74" name="undefined" type="group">
        <graphics fgcolor="#000000" bgcolor="#FFFFFF"
             type="rectangle" x="616" y="483" width="46" height="34"/>
        <component id="1"/>
```

```
        <component id="40"/>
    </entry>
```

Group OR:

```
     <entry id="51" name="hsa:10000 hsa:207 hsa:208" type="gene"
        link="http://www.kegg.jp/dbget-bin/www_bget?hsa:10000+hsa:207+hsa:208">
        <graphics name="AKT3, DKFZp434N0250, PKB-GAMMA, PKBG, ..." ...
             type="rectangle" x="616" y="408" width="46" height="17"/>
    </entry>
```

BioPax `owl` language for group AND and OR:

Group AND:

```
 <bp:complex rdf:ID="pid_m_213397" >
  <bp:ORGANISM rdf:resource="#Homo_sapiens" />
  <bp:DATA-SOURCE rdf:resource="#PID_DataSource" />
  <bp:NAME rdf:datatype="...">PP1-C/PP-1G/Glycogen</bp:NAME>
  <bp:COMPONENTS rdf:resource="#pid_x_203746" />
  <bp:COMPONENTS rdf:resource="#pid_x_203747_0S101" />
  <bp:COMPONENTS rdf:resource="#pid_x_203748" />
</bp:complex>
```

Group OR:

```
<bp:protein rdf:ID="pid_m_200318" >
  <bp:ORGANISM rdf:resource="#Homo_sapiens" />
  <bp:DATA-SOURCE rdf:resource="#PID_DataSource" />
  <bp:NAME rdf:datatype="...">FOS family</bp:NAME>
  <bp:XREF rdf:resource="#pid_b_200318_200480" />
  <bp:XREF rdf:resource="#pid_b_200318_201792" />
  <bp:XREF rdf:resource="#pid_b_200318_201793" />
  <bp:XREF rdf:resource="#pid_b_200318_201794" />
</bp:protein>
```

```
<bp:relationshipXref rdf:ID="pid_b_200318_200480">
  <bp:DB rdf:datatype="...">UniProt</bp:DB>
  <bp:ID rdf:datatype="...">P01100</bp:ID>
  <bp:RELATIONSHIP-TYPE rdf:datatype="...">protein family member</bp:RELATIONSHIP-TYPE>
</bp:relationshipXref>
<bp:relationshipXref rdf:ID="pid_b_200318_201792">
  <bp:DB rdf:datatype="...">UniProt</bp:DB>
  <bp:ID rdf:datatype="...">P15407</bp:ID>
  <bp:RELATIONSHIP-TYPE rdf:datatype="...">protein family member</bp:RELATIONSHIP-TYPE>
</bp:relationshipXref>
<bp:relationshipXref rdf:ID="pid_b_200318_201793">
  <bp:DB rdf:datatype="...">UniProt</bp:DB>
  <bp:ID rdf:datatype="...">P15408</bp:ID>
  <bp:RELATIONSHIP-TYPE rdf:datatype="...">protein family member</bp:RELATIONSHIP-TYPE>
</bp:relationshipXref>
<bp:relationshipXref rdf:ID="pid_b_200318_201794">
  <bp:DB rdf:datatype="...">UniProt</bp:DB>
  <bp:ID rdf:datatype="...">P53539</bp:ID>
  <bp:RELATIONSHIP-TYPE rdf:datatype="...">protein family member</bp:RELATIONSHIP-TYPE>
</bp:relationshipXref>
```


2 Supplementary material: compound mediated signals

KGML for compound mediated signals:


```
Direct definition:
  <entry id="52" name="hsa:23533 hsa:5290 hsa:5291  ..." type="gene"
        link="http://www.kegg.jp/dbget-bin/www_bget?hsa:23533+hsa:5290+hsa:5291+...">
        <graphics name="PIK3R5, F730038I15Rik, FOAP-2, P101-PI3K, p101..." ...
             type="rectangle" x="334" y="408" width="46" height="17"/>
```

```
    </entry>
    <entry id="62" name="hsa:5170" type="gene"
        link="http://www.kegg.jp/dbget-bin/www_bget?hsa:5170">
        <graphics name="PDPK1, MGC20087, MGC35290, PDK1, PRO0461" ...
             type="rectangle" x="516" y="408" width="46" height="17"/>
    </entry>
   <entry id="65" name="cpd:C05981" type="compound"
        link="http://www.kegg.jp/dbget-bin/www_bget?C05981">
        <graphics name="C05981" fgcolor="#000000" bgcolor="#FFFFFF"
             type="circle" x="421" y="408" width="8" height="8"/>
    </entry>

    <relation entry1="52" entry2="62" type="PPrel">
        <subtype name="compound" value="65"/>
        <subtype name="activation" value="--&gt;"/>
    </relation>
```

Indirect definition:

```
    <entry id="45" name="hsa:5139 hsa:5140" type="gene"
        link="http://www.kegg.jp/dbget-bin/www_bget?hsa:5139+hsa:5140">
        <graphics name="PDE3A, CGI-PDE..." fgcolor="#000000" bgcolor="#BFFFBF"
             type="rectangle" x="770" y="509" width="46" height="17"/>
    </entry>
    <entry id="23" name="hsa:5566 hsa:5567 hsa:5568  ..." type="gene"
        link="http://www.kegg.jp/dbget-bin/www_bget?hsa:5566+hsa:5567+hsa:5568+...">
        <graphics name="PRKACA, MGC102831, MGC48865, PKACA..." ...
             type="rectangle" x="884" y="509" width="46" height="17"/>
    </entry>
      <entry id="15" name="cpd:C00575" type="compound"
        link="http://www.kegg.jp/dbget-bin/www_bget?C00575">
        <graphics name="C00575" fgcolor="#000000" bgcolor="#FFFFFF"
```

```
            type="circle" x="824" y="510" width="8" height="8"/>
    </entry>

    <relation entry1="45" entry2="15" type="PCrel">
        <subtype name="inhibition" value="--|"/>
    </relation>
       <relation entry1="15" entry2="23" type="PCrel">
        <subtype name="activation" value="--&gt;"/>
    </relation>
```

Toy example of the BioPax `owl xml` language for compound mediated signals:

```
======== PATHWAY COMPONENTS  =======

<bp:PATHWAY-COMPONENTS rdf:resource="Z" />
<bp:PATHWAY-COMPONENTS rdf:resource="Y" />

============ PHYSICAL ENTITY =========

<bp:complex rdf:ID="A" >
  <bp:ORGANISM rdf:resource="#Homo_sapiens" />
  <bp:DATA-SOURCE rdf:resource="... />
  <bp:NAME rdf:datatype="...">AA</bp:NAME>
  <bp:COMPONENTS rdf:resource="B1" />
  <bp:COMPONENTS rdf:resource="C1" />
</bp:complex>
<bp:smallMolecule rdf:ID="B" >
  <bp:DATA-SOURCE rdf:resource="..." />
  <bp:NAME rdf:datatype="...">BB</bp:NAME>
  <bp:XREF rdf:resource="#ChemicalAbstracts_..." />
</bp:smallMolecule>
```

```
<bp:smallMolecule rdf:ID="C" >
  <bp:DATA-SOURCE rdf:resource="..." />
  <bp:NAME rdf:datatype="...">CC</bp:NAME>
  <bp:XREF rdf:resource="#ChemicalAbstracts_..." />
</bp:smallMolecule>
<bp:protein rdf:ID="D" >
  <bp:ORGANISM rdf:resource="#Homo_sapiens" />
  <bp:DATA-SOURCE rdf:resource="..." />
  <bp:NAME rdf:datatype="...">DD</bp:NAME>
  <bp:XREF rdf:resource="#UniProt_..." />
</bp:protein>
<bp:protein rdf:ID="E" >
  <bp:ORGANISM rdf:resource="#Homo_sapiens" />
  <bp:DATA-SOURCE rdf:resource="..." />
  <bp:NAME rdf:datatype="...">EE</bp:NAME>
  <bp:XREF rdf:resource="#UniProt_..." />
  <bp:SYNONYMS rdf:datatype="...">
FF</bp:SYNONYMS>
</bp:protein>
<bp:protein rdf:ID="F" >
  <bp:ORGANISM rdf:resource="#Homo_sapiens" />
  <bp:DATA-SOURCE rdf:resource="..." />
  <bp:NAME rdf:datatype="...">FF</bp:NAME>
  <bp:XREF rdf:resource="#UniProt_..." />
  <bp:SYNONYMS rdf:datatype="...">FFF</bp:SYNONYMS>
</bp:protein>

==== PHYSICAL ENTITY PARTICIPANTS =======

<bp:physicalEntityParticipant rdf:ID="A1" >
  <bp:PHYSICAL-ENTITY rdf:resource="A" />
```

```
</bp:physicalEntityParticipant>
<bp:sequenceParticipant rdf:ID="B1" >
  <bp:PHYSICAL-ENTITY rdf:resource="B" />
</bp:sequenceParticipant>
<bp:physicalEntityParticipant rdf:ID="C1" >
  <bp:PHYSICAL-ENTITY rdf:resource="C" />
<bp:physicalEntityParticipant rdf:ID="D1" >
  <bp:PHYSICAL-ENTITY rdf:resource="D"
</bp:sequenceParticipant>
<bp:sequenceParticipant rdf:ID="E1" >
  <bp:PHYSICAL-ENTITY rdf:resource="E" />
</bp:sequenceParticipant>
<bp:sequenceParticipant rdf:ID="F1" >
  <bp:PHYSICAL-ENTITY rdf:resource="F" />
</bp:sequenceParticipant>

========== INTERACTIONS ======

<bp:biochemicalReaction rdf:ID="X" >
  <bp:DATA-SOURCE rdf:resource="" />
  <bp:EVIDENCE>
    <bp:evidence rdf:ID="">
      <bp:EVIDENCE-CODE rdf:resource="" />
    </bp:evidence>
  </bp:EVIDENCE>
  <bp:XREF rdf:resource="#Pubmed..." />
  <bp:XREF rdf:resource="#Pubmed..." />
  <bp:LEFT rdf:resource="A1" />
  <bp:RIGHT rdf:resource="C1" />
  <bp:RIGHT rdf:resource="D1" />
</bp:biochemicalReaction>
```

```
<bp:biochemicalReaction rdf:ID="Y" >
  <bp:DATA-SOURCE rdf:resource="" />
  <bp:EVIDENCE>
    <bp:evidence rdf:ID="">
      <bp:EVIDENCE-CODE rdf:resource="" />
    </bp:evidence>
  </bp:EVIDENCE>
  <bp:XREF rdf:resource="#Pubmed..." />
  <bp:XREF rdf:resource="#Pubmed..." />
  <bp:LEFT rdf:resource="E1" />
  <bp:LEFT rdf:resource="C1" />
  <bp:RIGHT rdf:resource="F1" />
</bp:biochemicalReaction>
```

3 Supplementary material: an extract for the KGML correspondent to insulin signalling pathway

```
Entries definition:
    <entry id="3" name="hsa:3635" type="gene"
        link="http://www.kegg.jp/dbget-bin/www_bget?hsa:3635">
        <graphics name="INPP5D, MGC104855, MGC142140, MGC142142, ..." ...
             type="rectangle" x="334" y="220" width="46" height="17"/>
    </entry>
    <entry id="2" name="hsa:51763" type="gene"
        link="http://www.kegg.jp/dbget-bin/www_bget?hsa:51763">
        <graphics name="INPP5K, PPS, SKIP" fgcolor="#000000" bgcolor="#BFFFBF"
             type="rectangle" x="334" y="241" width="46" height="17"/>
    </entry>
    <entry id="62" name="hsa:5170" type="gene"
        link="http://www.kegg.jp/dbget-bin/www_bget?hsa:5170">
```

```
    <graphics name="PDPK1, MGC20087, MGC35290, PDK1, PRO0461" ...
         type="rectangle" x="516" y="408" width="46" height="17"/>
</entry>
<entry id="51" name="hsa:10000 hsa:207 hsa:208" type="gene"
    link="http://www.kegg.jp/dbget-bin/www_bget?hsa:10000+hsa:207+hsa:208">
    <graphics name="AKT3, DKFZp434N0250, PKB-GAMMA, PKBG, ..." ...
         type="rectangle" x="616" y="408" width="46" height="17"/>
</entry>
<entry id="45" name="hsa:5139 hsa:5140" type="gene"
    link="http://www.kegg.jp/dbget-bin/www_bget?hsa:5139+hsa:5140">
    <graphics name="PDE3A, CGI-PDE..." fgcolor="#000000" bgcolor="#BFFFBF"
         type="rectangle" x="770" y="509" width="46" height="17"/>
</entry>
<entry id="23" name="hsa:5566 hsa:5567 hsa:5568  ..." type="gene"
    link="http://www.kegg.jp/dbget-bin/www_bget?hsa:5566+hsa:5567+hsa:5568+...">
    <graphics name="PRKACA, MGC102831, MGC48865, PKACA..." ...
         type="rectangle" x="884" y="509" width="46" height="17"/>
</entry>
<entry id="65" name="cpd:C05981" type="compound"
    link="http://www.kegg.jp/dbget-bin/www_bget?C05981">
    <graphics name="C05981" fgcolor="#000000" bgcolor="#FFFFFF"
         type="circle" x="421" y="408" width="8" height="8"/>
</entry>
<entry id="15" name="cpd:C00575" type="compound"
    link="http://www.kegg.jp/dbget-bin/www_bget?C00575">
    <graphics name="C00575" fgcolor="#000000" bgcolor="#FFFFFF"
         type="circle" x="824" y="510" width="8" height="8"/>
</entry>
<entry id="52" name="hsa:23533 hsa:5290 hsa:5291  ..." type="gene"
    link="http://www.kegg.jp/dbget-bin/www_bget?hsa:23533+hsa:5290+hsa:5291+...">
    <graphics name="PIK3R5, F730038I15Rik, FOAP-2, P101-PI3K, p101..." ...
```

```
            type="rectangle" x="334" y="408" width="46" height="17"/>
    </entry>
```


Relation definition:


```
    <relation entry1="3" entry2="65" type="PCrel">
        <subtype name="inhibition" value="--|"/>
    </relation>
    <relation entry1="2" entry2="65" type="PCrel">
        <subtype name="inhibition" value="--|"/>
    </relation>
    <relation entry1="62" entry2="51" type="PPrel">
        <subtype name="activation" value="--&gt;"/>
        <subtype name="phosphorylation" value="+p"/>
    </relation>
    <relation entry1="51" entry2="45" type="PPrel">
        <subtype name="activation" value="--&gt;"/>
        <subtype name="phosphorylation" value="+p"/>
    </relation>
    <relation entry1="45" entry2="15" type="PCrel">
        <subtype name="inhibition" value="--|"/>
    </relation>
    <relation entry1="15" entry2="23" type="PCrel">
        <subtype name="activation" value="--&gt;"/>
    </relation>
    <relation entry1="52" entry2="62" type="PPrel">
        <subtype name="compound" value="65"/>
        <subtype name="activation" value="--&gt;"/>
    </relation>
```
